# Birth Registry of Women With Systematic Lupus Erythematosus: The Greek Experience

**DOI:** 10.31138/mjr.29.4.228

**Published:** 2018-12-18

**Authors:** Stella Ntali, Lamprini Pantazi, Kyriaki Mpoki, Dionysios Nikolopoulos, Antonis Fanouriakis, Ioannis Kallitsakis, Charalambos Papagoras, Despoina Dimopoulou, Eleni Kteniadaki, Evgenia Emmanouilidou, Vassiliki Chania, George Bertsias

**Affiliations:** 1 Private Practice Rheumatologist, Thessaloniki, Greece,; 2 Rheumatology Unit, GHA, Sismanogleio, Athens, Greece,; 3 Rheumatology and Clinical Immunology Unit, 4^th^ Internal Medicine University Clinic, GUH, Attikon, Athens, Greece,; 4 Private Practice Rheumatologist, Chania, Crete, Greece,; 5 1^st^ University Internal Medicine Clinic, Medical School, DUT, Alexandroupolis, Greece,; 6 4^th^ Internal Medicine Clinic, AUTH, Ippokrateio GHTH, Thessaloniki, Greece,; 7 Private Practice Rheumatologist, Herakleion, Crete, Greece,; 8 Rheumatology and Clinical Immunology and Allergiology Clinic, PAGNI, Herakleion, Crete, Greece

**Keywords:** SLE, pregnancy, Registry, birth outcome

## Abstract

Pregnancy in women with SLE (Systematic Lupus Erythematosus) is considered of “high risk” since it has been related with adverse events both in the mother and the foetus. Many studies have reported relapse of the disease during the pregnancy and the first trimester post-labour, while others have found no difference in terms of frequency and type of relapses. Moreover, adverse obstetrical events like recurrent pregnancy loss, preeclampsia, prematurity, intrauterine growth restriction and neonatal lupus syndrome tend to occur more often in patents with SLE. However, most of these data regarding the burden and pregnancy outcomes in SLE come from retrospective studies of previous decades, and in non-Caucasian patients. To this end, more recent studies have suggested overall improved outcomes of pregnancy, still their results are often conflicting. The purpose of this study is to record, through a prospective observational (non-interventional) study, the contemporary prognosis of pregnancy in women with SLE who are followed-up by private and hospital physicians in Greece. In particular, we aim to establish a registry to study the course of the disease during pregnancy, the outcome of pregnancy and the possible negative or positive prognostic factors, the effect of drugs on pregnancy and the foetus.

## INTRODUCTION

Systemic Lupus Erythematosus primarily affects women of reproductive age in whom pregnancy is a frequent and anticipated event. Pregnancy in these women is generally considered as “high risk”, as it is associated with an increased incidence of complications in the mother and foetus. Therefore, many non-specialist doctors and health professionals often discourage SLE patients from childbirth. However, the prevention of these complications and their management have improved significantly over the last decades.^1^ During this time, our knowledge of the pathogenesis and treatment of the disease, as well as the course of the disease during pregnancy, the possible complications and the implicated risk factors have been greatly enhanced. During pregnancy in women with SLE, important parameters should be considered, such as: (a) the effect of pregnancy on the disease activity and its progression; (b) the effect of the disease and its treatment in the foetus; and (c) maternal health during pregnancy and postpartum. Several studies have dealt with the above parameters, and have found a reduced rate of successful pregnancy in SLE patients with high disease activity, increased maternal mortality compared to the general population and increased pregnancy morbidity such as preterm labour, preeclampsia and caesarean section.^2–4^ In addition, women with SLE have more frequent automatic miscarriages, thromboembolism and postpartum infection compared to the general population.

As far as embryos are concerned, SLE has been associated with prematurity, small birth embryos and an increased number of embryos requiring hospitalization in the neonate unit.^2,5^ More reassuring are the results from the prospective study PROMISSE, in which 80% of women with SLE had a good outcome.^6^ Finally, with regard to the progression of SLE during pregnancy, the results from several small studies are contradictory. In some of these, that matched pregnant Lupus patients to non-pregnant women, stable disease activity was found. However, more recent studies demonstrated a two-or three-fold increase in SLE activity during pregnancy. In these studies, the risk for measurable disease activity ranged between 40–50%. These differences may be due to the heterogeneity of the studied patient populations for the severity of the disease but also to the way in which the disease activity is determined.^7^

In conclusion, existing data is based largely on studies of previous decades, retrospective, with contradictory results. Despite increasing experience and better knowledge-awareness of physicians dealing with specific cases (gynaecologists and rheumatologists), there are reports of an increased incidence of complications during pregnancy. On the basis of the above, it is important to have a contemporary, systematic record of the Greek experience in the pregnancies of SLE patients.

## RESEARCH OBJECTIVE – METHODOLOGY

This is a prospective, multicentre, non-intrusive observation study lasting three (3) years. During this time, women diagnosed with SLE who are pregnant will be monitored, with the consent of the responsible Rheumatologist in collaboration with the pregnant gynaecologist. A structured questionnaire will be used for monitoring (*Table 1*). Monitoring will be done at the start of pregnancy (positive pregnancy test) and every 3 months or earlier, depending on the course of the disease and pregnancy and for the first year after childbirth. The study will be supervised and coordinated by the Clinic of Rheumatology, Clinical Immunology and Allergiology of PAGNI and the Rheumatology-Clinical Immunology Unit of Attiki, the 4th Clinic of Pathology in Ippokrateio, Thessaloniki, the Clinical Immunology-Rheumatology Unit 2nd Pathological Clinic, Ippokrateio, Democritus University and Rheumatologists. In the pre-defined questionnaire, information is recorded for:

- Demographics (age, education, habits [smoking], body mass index)
- Disease manifestations and classification criteria met by patients (ACR 1997 and / or SLICC 2012 criteria)
- Obstetrical history
- Information on this pregnancy (physical conception, assisted, disease activity and medication at conception)

**Table 1.** Study parameters evaluated by structured forms.

Demographics	[more specific: year of birth? Ethnicity? Etc.
Symptoms of the disease	ACR1997, SLICC criteria
Personal history, drug (related to disease) history	
Obstetrical history	Previous pregnancies, pregnancy outcome, miscarriages, prematurity, disease relapse, preeclampsia
Immunological profile during pregnancy	APS, antiRo, La, anti-dsDNA
Use of medications during pregnancy and 12 months after labour	
Disease activity at the time of conception, during pregnancy and during 12 months after labour	SLEDAI-2K PGA
Pregnancy outcome	Live birth, miscarriage, prematurity, complications preeclampsia, diabetes, disease relapse weight of birth, week, kind of labour, gender, APGAR score

Each visit records:

- clinical findings of the patient (signs of disease: activity, arthritis, rashes, mouth ulcers, hair loss, myositis) including Blood Pressure
- findings from laboratory testing every three months (general blood, hepatic and renal function, urinary output, 24-hour urine collection, uric acid, but also
- immunoassay - titre of anti-ds-DNA, C3 / C4, titre of anti-Ro/SSA, anti-La/SSB ant antiphospholipid antibodies (anti-cardiolipin IgG/IgM, anti-β2-glycoprotein I IgG/IgM, lupus anticoagulan will be done before or with confirmation of pregnancy.

With the above findings, it will be possible: a) to calculate the SLEDAI-2K (SLE Disease Activity Index 2000) index every 3 months and the course of the disease during pregnancy and the first year after birth; b) to record the medication received by the patient at the beginning of and during pregnancy.

At the end of pregnancy:

- the type of birth (normal or caesarean, spontaneous or programmed)
- gender, birth weight, birth week, APGAR score of the newborn, as well as other possible findings related to it such as congenital abnormalities or neonatal lupus findings.

The above data will be entered into a secure, specially configured electronic database that is installed in the Rheumatology Clinic on a protected server and the PAGNI network. The operation and maintenance of the database is strictly supervised by the scientifically accountable protocol, and access is granted only to authorized users / researchers. All principles of anonymity, confidentiality and non-traceability of data are adhered to.

### Subjects of Methodology and Ethics

- The study is a non-intrusive, prospective observational study. It does not in any case cost the patient or hospital (PAGNI), as it includes the planned monitoring and care (including any laboratory tests) of SLE patients and pregnancy in the context of everyday clinical practice in accordance with the European Rheumatic Society Guidelines.
- The timetable for the study is 36 months (3 years). In order to ensure the extraction of statistically significant results, the study is expected to include 100 SLE patients.
- The participation of the patients is a strictly voluntarily-followed, detailed briefing by a member of the research team with written consent. The research protocol and consent form have been approved by the Ethics Committees of the participating parties. All therapies, laboratory investigations, and general care of patients participating in the study are determined by the attending physician and patient involvement in the study does not in any way affect the above.
- At any time during the study, if a patient wishes to discontinue her participation in the study, this can be done by direct communication with the Principal Investigator of the study (“right to withdraw”). This will be accompanied by destruction of personal data / data from study samples.

### Suitability of the research team

The research team consists of clinicians, scientific associates and specialized nurses with significant experience in systematically recording demographic, clinical and other data from patients with chronic inflammatory rheumatic diseases, organizing a registry, according to the objectives of the study. The polycentric nature of the study is considered necessary to collect data from a sufficient number of women with SLE.

## IMPORTANCE OF THE STUDY AND EXPECTED BENEFITS

The purpose of the study is to record the Greek experience with pregnancies in mothers with SLE and their prognosis. To date, there is no corresponding prospective study in Greece. The results of the study will serve to assess the risk of developing complications in the mother-patient and the foetus, thus allowing more accurate information to patients and their relatives or associates that are unfortunately often receiving false data and information. The Greek health system differs from those in other countries in that a large proportion of patients are tended to by private doctors, without always having the support of a specialized centre. This study will also involve individuals’ private practitioners, and their experience will be recorded. We also have the opportunity to monitor the course of the disease during pregnancy and the first year after that, and to detect specific prognostic factors for the occurrence of events or poor outcomes.

## BUDGET AND FUNDING BODY

- The research budget is €7,000 and is distributed as follows: a) maintenance of a patient database for the registration of patient data: €3,000; b) support research personnel for data entry & statistical processing: €3,000; and c) supplies-stationery: €1,000.
- The scientists of the study will have no financial gain.
- The funding of the study comes from the Hellenic Rheumatological Society & Professional Association of Rheumatologists (number 654).
